# LB1532. The Impact of COVID-19 on nursing home residents’ clinical, functional, and psychosocial outcomes

**DOI:** 10.1093/ofid/ofac492.1878

**Published:** 2022-12-15

**Authors:** Lori Popejoy, Amy Vogelsmeier, Gregory Petroski, Daivd Mehr, Marilyn Rantz, Steven Miller, Christelle Ilboudo, Justina Yevu Johnson, Michelle Dardis

**Affiliations:** University of Missouri, Columbia, MO; University of Missouri, Columbia, MO; University of Missouri, Columbia, MO; University of Missouri, Columbia, MO; University of Missouri, Columbia, MO; University of Missouri, Columbia, MO; Children's Mercy, Kansas City, Missouri; University of Missouri, Columbia, MO; University of Missouri, Columbia, MO

## Abstract

**Background:**

Data on COVID-19 related nursing home infections and mortality accumulated at a rapid pace; yet little is known about the impact of nursing homes’ response to COVID-19 on resident clinical, functional, and psychosocial outcomes.

**Methods:**

We examined aggregated Minimum Data Set (MDS) assessments to describe resident outcomes using an interrupted times series methodology for three timeframes: pre-COVID (1/2019 to 2/2020), pandemic (3/2020–12/2021), and vaccination (1/2021-6/2021). Data included 307,558 federally mandated resident MDS assessments from 60,846 resident in 489 nursing homes in a Mid-Western state. We calculated MDS based quality measures (QM) using definitions available from Centers for Medicare and Medicaid Services. Each QM-based outcome was fit to a logistic regression model using the method of generalized estimating equations.

**Results:**

None of the QMs displayed a statistically significant trend pre-COVID. The prevalence of excessive weight loss and ADL decline increased sharply during the pandemic and reversed that trend with vaccination. Pressure ulcers among high-risk residents followed a similar trend, although pandemic and vaccination-related regression parameters for that QM were only marginally significant (p = .08). Pain worsened during the pandemic and vaccination period approaching significance (p=.07). Antipsychotic medication use worsened in the pandemic (p< .001) and did not improve in the vaccination period. Other QMs including any fall, fall with major injury, and incontinence did not exhibit statistically significant change in trend.

Prevalence Profiles

**Figure:**
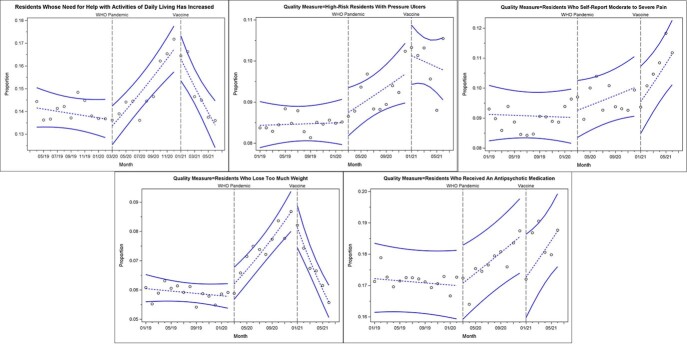

Circles: Observed proportions, Dashed Line: Model expected value, Solid Lines: 95% confidence limits for expected values

**Conclusion:**

We noted significant changes in QMs for antipsychotic use, ADL loss, and weight loss, with the latter two improving in the vaccination period. Isolation, disease outbreaks, and staffing issues in facilities could have affected these QMs. Data variability may have limited our ability to detect other changes. Antipsychotics may have increased with the need to reduce wandering and other behaviors common in the nursing home population; behaviors high risk for spreading COVID-19. Why antipsychotic use did not improve during the vaccination period is less clear. Data beyond June of 2021 may help clarify the pattern of antipsychotic use.

**Disclosures:**

**Lori Popejoy, PhD, RN, FAAN**, New Path: Board Member|New Path: Ownership Interest **Amy Vogelsmeier, PhD, RN, FAAN**, New Path: Board Member|New Path: Ownership Interest **Marilyn Rantz, PhD, RN, FAAN**, New Path: Board Member|New Path: Ownership Interest.

